# Natural history and management of small renal masses

**DOI:** 10.3747/co.v16i0.409

**Published:** 2009-05

**Authors:** T. Abou Youssif, S. Tanguay

**Keywords:** Renal cell carcinoma, treatment, outcome

## Abstract

The recent stage migration observed for renal tumours is contributing to a significant increase in the diagnosis of small renal masses. This evolution has led to a significant change in the approach to renal masses. New options such as observation or energy ablation are gaining popularity in a subset of this patient population. In addition, the observed changes directly contribute to the increased use of nephron-sparing surgery. A better understanding of the various characteristics of these masses will allow for a better understanding of the natural history of these masses and for selection of the optimal therapeutic approach.

## 1. INTRODUCTION

Small renal masses (srms) are defined as cortical renal masses smaller than 4 cm in diameter. An observed stage migration is contributing to the increased detection of srms. Between 1983 and 2002, the incidence of renal tumours 2–4 cm in diameter more than tripled to 3.3 from 1.0 cases per 100,000 1. In the 1970s, approximately 10% of renal cell carcinomas (rccs) were discovered incidentally, as compared with 60% in 1998 2.

Increased detection of asymptomatic srms cannot be fully explained by the more frequent use of abdominal imaging. Mindrup *et al*. compared the frequency of detection of occult rcc at autopsy for the periods 1955–1960 and 1991–2001, and noticed that, despite the recent use of more frequent and sophisticated imaging techniques, no difference was evident in the frequency of occult rcc (0.4% and 0.5% respectively) 3. In that series, the mean size of the occult rcc detected at autopsy in the earlier period was about one third the size of the tumours detected in the more recent group (1.65 cm vs. 4.63 cm) 3.

Peak rcc incidence occurs in patients more than 65 years of age 4. Patients in that age group often have significant medical comorbidities that might affect the choice of therapeutic options.

Management of srms must be individualized, because several patient- and tumour-related factors affect management strategy. The identification of these parameters necessitates revisiting the natural history of srms and the safety and efficacy of the various available treatment modalities.

## 2. NATURAL HISTORY OF SRMs

Understanding the biologic behaviour and natural history of srms is crucial in predicting tumour growth and metastatic potential, so as to properly select the methods and optimal timing of intervention.

### 2.1 Pathologic Features

A significant number of srms will prove to be benign. Vasudevan *et al*. reported a 33% benign histology in 70 renal masses smaller than 5 cm 5. Similarly, a report of 100 laparoscopic partial nephrectomies for a mean tumour size of 2.9 cm showed that 32% of the surgeries were performed for benign disease, as indicated by the final pathology specimen 6.

The common perception that srms are always indolent tumours is not completely true. Of renal masses smaller than 4 cm, 20%–30% are reported to be aggressive tumours with highly metastatic potential, even if dimensionally small 7,8. Other reported series identified aggressive pathology features in 25%–38% of srms 7,9. In addition, Hsu *et al*. also demonstrated extracapsular extension in 38% of 50 resected rccs smaller than 3 cm 7.

Lesions between 3 cm and 4 cm in diameter are most likely to have aggressive pathologic features. Perinephric fat invasion is found in 4.2% of tumours smaller than 2 cm, 14.9% of tumours 2.1–3 cm, and 35.7% of tumours 3.1–4 cm 9. The Fuhrman grade of small rcc tumours is low in most cases; only 5%–6.5% of tumours 2–3 cm and 18.7%–25.5% of tumours 3–4 cm have a high Fuhrman grade (3 or 4) 9,10.

As indicated earlier, srms show heterogeneous pathologic features ranging from totally benign to highly aggressive, and hence determination of the exact nature of an incidentally discovered mass is crucial before a treatment decision is made.

### 2.2 Progression

Although the growth rates of srms can vary considerably, a recent meta-analysis of clinically localized tumours determined an overall median growth rate of 0.28 cm annually for lesions under active surveillance at a mean follow-up of 34 months (median: 32 months; range: 26–39 months) 4. Moreover, Kunkle *et al*. reported that 26%–33% of incidentally detected renal masses demonstrate zero net growth when observed 11.

The aggressive potential of small renal masses was studied. A recent review revealed an association with tumours 3.0 cm or smaller in up to 5% of patients with synchronous rcc metastases 12. Minardi *et al*. evaluated 48 patients with pT1a clear-cell rcc who underwent nephron-sparing surgery (nss). Of these 48 patients, 3.9% died of metastatic renal cancer at a median follow-up of 2 years 13. Distant metastases occurred in 2.4% of patients with tumours smaller than 3 cm as compared with 8.4% of patients with tumours between 3.1 cm and 4 cm 9. This information clearly supports the variable and occasionally unpredictable behaviour of srms.

### 2.3 Predictors of Progression

To properly select the appropriate treatment strategy, it would be helpful to establish clinical and pathologic predictors of future tumour growth or progression to metastatic disease. Initial tumour diameter, tumour growth rate, and age have been examined with regard to patient outcomes.

#### 2.3.1 Initial Tumour Size

Tumour size at presentation was found not to correlate with growth rate in the meta-analysis published by Chawla *et al*., nor in our recently published series 4,14. However, tumour size is a significant clinical predictor for the presence of biopsy-proven synchronous metastatic rcc. Kunkle *et al*. reported a 22% increased in the odds of synchronous metastasis with each centimetre increase in tumour size 12.

#### 2.3.2 Growth Rate

The behaviour of srms remains difficult to predict. Kunkle *et al*. compared radiographic and pathologic characteristics of enhancing renal masses under active surveillance with zero net radiographic growth to those with positive growth 11. Pathology assessment confirmed the same incidence of malignancy in tumours with either zero growth rate or with a positive growth rate (83% and 89% respectively, *p* = 0.56), suggesting that growth rate is not indicative of malignant or benign histology 11.

#### 2.3.3 Age

In the observational study by Kouba *et al*. of renal masses, patients under 60 years of age had a trend toward a more rapid rate of tumour growth than did patients 60 years of age and older (0.90 cm vs. 0.60 cm annually, *p* = 0.0570) 15. Although the rationale for observation appears to be safe in selected older patients, younger patients tend to have faster-growing tumours, supporting treatment at diagnosis because of the potential for progression.

## 3. DIAGNOSIS OF SRMs

Accurate differentiation of srms between categories of benign and malignant histology is the cornerstone of the treatment decision.

### 3.1 Imaging and Pathology

Benign lesions such as simple cysts and angiomyolipomas usually have classic imaging features allowing for an accurate diagnosis. However, oncocytomas and occasionally low-fat angiomyolipomas are often difficult to differentiate from rccs with current imaging modalities. Remzi *et al*. found that only 17% of all benign lesions were correctly identified as benign on preoperative computed tomography (ct) imaging, and that 43% of patients who were assessed incorrectly on preoperative ct underwent unnecessary radical surgery 16.

### 3.2 Initial Tumour Size

Evaluation of 1208 srms confirmed a prevalence of benign lesions of 15%, 14%, and 14% in the tumour size ranges of 0.1–1.0 cm, 1.1–2.0 cm, and 2.1–3.0 cm respectively. However, the incidence of benign lesions decreased significantly in tumours measuring 3.1–4.0 cm (8%, *p* = 0.001) 17. Similarly, Frank *et al*. reviewed the pathology specimens of 2770 patients and found that each 1 cm increase in tumour size was associated with a 17% increase in the odds of malignancy 10.

Two recent studies attempted to correlate pathology characteristics and tumour diameter 9,18. Remzi *et al*. studied 287 tumours smaller than 4 cm in diameter and found a significant increase in the probability of multifocality, higher nuclear grade, type 2 papillary rcc, advanced stage, and distant metastases with increase in diameter 9. Pahernik *et al*. also observed a sharp increase in the incidence of adverse prognostic parameters in tumours greater than 3 cm 18.

### 3.3 Tumour Growth Rate

Tumour growth rate is not an ideal parameter for predicting tumour histology. Chawla *et al*. demonstrated no statistical difference in mean tumour size at diagnosis (2 cm vs. 2.2 cm) or mean growth rate (0.16 cm vs. 0.35 cm annually) between oncocytomas and rcc under observation 4.

### 3.4 Preoperative Nomogram

In an effort to help predict preoperative histology, a nomogram was created. After analysis of 862 srms, the probability of benign—or likely indolent—pathology findings was modeled using a multivariable logistic regression model based on age, sex, radiographic tumour size, symptoms at presentation, and smoking history. According to this nomogram, srms in older men and younger women are more likely to be benign 19.

### 3.5 Role of Renal Biopsy

Imaging alone being unable to reliably define the nature of srms, histologic confirmation can be obtained by percutaneous biopsy. Percutaneous needle biopsy remains underutilized by urologists because of a perception of unreliability. However, contemporary use of renal biopsy has proved to be reliable in most patients.

Renal biopsy could help to select the optimal treatment strategy and possibly to avoid nephrectomy for benign lesions. In a recent study, biopsy with an 18-gauge needle was performed for 235 masses. Of these masses, 184 had an appropriate diagnostic. The accuracy of the biopsy result was confirmed in 108 renal surgeries, confirming a 100% biopsy accuracy rate in distinguishing malignant from benign lesions and a 98% ability to determine the histologic tumour type 20. Neuzillet *et al*. reported on 88 biopsies performed in solid srms. Histopathologic tumour type was accurately identified in 92%, and Fuhrman nuclear grade, in 69.8% 21.

The identification of diagnostic and prognostic molecular markers to be evaluated in the renal biopsy is underway and will hopefully help to define the rccs that are likely to exhibit aggressive behaviour.

## 4. TREATMENT OPTIONS

A variety of treatment options for patients with srms have evolved over the years. These options range from surgical excision, through ablative techniques, to active surveillance.

### 4.1 Surgical Excision

Surgical excision remains the “gold standard” for the management of srms. The improved understanding of the effect of radical nephrectomy in promoting the development of chronic kidney disease reinforces the importance of nss when feasible. As compared with partial nephrectomy, radical nephrectomy has been associated with an increase in long-term mortality [risk ratio (rr): 2.16; *p* = 0.02] 22. Moreover, partial nephrectomy has also been associated with a better health-related quality of life secondary to preservation of renal function and of overall quality of life 23.

Partial nephrectomy has proved to be a surgical approach associated with low morbidity and high patient satisfaction that also provides excellent oncologic and functional outcomes at 10 years 24. The acceptance of elective nss for tumours smaller than 4 cm and also potentially for all tumours smaller than 7 cm should lead to an increase in the number of nsss performed 25.

In an effort to minimize morbidity, laparoscopic partial nephrectomy has emerged as a minimally invasive approach that is technically feasible in experienced hands 26. Gill *et al*. compared outcomes in 1800 patients undergoing open partial nephrectomy by experienced surgeons with the initial experience with laparoscopic partial nephrectomy in patients with a single renal tumour smaller than 7 cm 27. Laparoscopic partial nephrectomy was associated with a shorter operative time, reduced operative blood loss, and a shorter hospital stay 27. Laparoscopic partial nephrectomy was, however, associated with longer warm ischemia time. For patients with a solitary T1N0M0 rcc, 3-year cancer-specific survival was 99.3% and 99.2% after laparoscopic and open partial nephrectomy respectively 27. A recently published report of oncologic and renal functional outcomes 5 years after laparoscopic partial nephrectomy showed excellent results, comparable to those of open nss 28.

### 4.2 Thermal Ablation

Minimally invasive ablative techniques using various forms of energy to ablate renal tumours, such as radiofrequency ablation or cryotherapy are also gaining acceptance 29.

#### 4.2.1 Cryotherapy

Cryoablation can be performed either percutaneously or in a laparoscopic approach; however, the percutaneous approach may be associated with inferior outcomes as compared with a standard laparoscopic approach 30.

During therapy, the ice ball created by the cryoprobes can be monitored in real time by ultrasound, magnetic resonance imaging, or laparoscope to ensure that the intended area is fully treated 31. The maximum recommended lesion size for cryoablation of rcc is 4.0 cm. Potential complications of cryoablation include hypothermic damage to normal tissue adjacent to the tumour, injury to adjacent organs, structural damage along the probe track, and development of secondary tumours if cancer cells are seeded during probe removal 31.

Cryoablation has proved to be oncologically effective. Gill *et al*. reported 3-year results for 56 patients undergoing laparoscopic renal cryoablation. At 3 years, cryolesions were 75% smaller, and 38% had completely disappeared 32. Postoperative needle biopsy found local recurrence in only 2 patients 32. Longer-term oncologic data are required to validate renal cryoablation as a treatment modality for patients with srms.

Recently a novel approach using sparc (single-port access renal cryoablation) for srms was proposed 33. In this technique, the entire cryoablation procedure is performed laparoscopically through a single port. The ability to monitor the progression of the ice ball with laparoscopic ultrasound, the ability to directly follow the development of the ice ball, and the opportunity to properly control any post-treatment bleeding from the site of renal puncture can be considered the most attractive features of this novel approach.

#### 4.2.2 Radiofrequency Ablation

Radiofrequency ablation (rfa) uses alternating electric current to heat tissue, causing cell death and ischemia leading to coagulative necrosis. The application of radiofrequency produces resistive friction that is converted into heat in the tissue. Heating tissues above 50°C induces cellular destruction and protein denaturation 31. The primary advantage of rfa over cryosurgery is its easier application: rfa is less cumbersome, uses smaller probes, and is a portable technique. However, with the currently available rfa technology, real-time monitoring of the treated area remains difficult 32. Kunkle and colleagues demonstrated in a meta-analysis that, as compared with cryoablation, rfa is associated with a high risk of local progression and a higher risk of the need for a repeat ablation.

### 4.3 Active Surveillance

The practice of active surveillance is contrary to all conventional treatment standards, which advocate early surgical removal of all suspected renal malignancies. Yet, available data on untreated renal tumours suggest that most srms tend to have a long natural history associated with slow growth rate and limited risk of metastasis, suggesting that in selected patients, treatment can often safely be delayed 34. Recently, Crispen *et al*. reported on outcomes in 82 patients with 87 srms who had a median of 14 months’ delay (mean: 21 months; range: 6–97 months) in their management 35. Overall, 66 of 87 tumours (76%) were surgically removed. Local disease recurrence was noted in the remnant kidney in only 1 of 73 patients (1%) with pathology-confirmed rcc. The estimated 1-year and 3-year cancer recurrence–free survival rates for these patients were 100% and 99%. No cancer-related deaths occurred, and no patient developed metastatic disease before or after intervention 35.

Many studies have evaluated outcomes in patients undergoing active surveillance for their srms 4,14,34–42. Indications for observation included one or a combination of the presence of significant comorbidities, patient choice, potential need for postoperative dialysis, a tumour in a solitary kidney, or bilateral renal tumours 4,34–39,42. All published series demonstrated a limited growth rate during the observation period and a low risk of metastasis in the absence of tumour growth.

We previously reported our initial experience in the observation of 24 patients with srms. After an average of 32 months of observation, tumour growth was demonstrated in 5 patients, and no patients developed metastatic disease during the observation period 36.

In a recent meta-analysis, Chawla *et al*. analyzed the published experience with 286 srms followed conservatively 4. Mean tumour size at presentation was 2.6 cm (median: 2.48 cm). Patients were followed for a mean of 34 months (median: 32 months; range: 26–39 months). Mean and median annual growth rates of 0.28 cm were observed 4. Progression to metastatic disease was reported in only 3 patients representing 1% of the entire patient cohort followed. The authors concluded that, under observation, most enhancing srms will grow at a slow rate. Initial and serial radiographic evaluation remain unable to predict the likelihood of progression for these lesions 4.

Recently, we published our updated experience in active surveillance for srms 14. We followed 35 patients for a mean of 44.3 months. Patients had tumours with a median diameter of 2.1 cm at the time of diagnosis. The mean annual size-growth rate of the tumours was 0.21 cm, and the mean and median annual volume-growth rates were 2.7 cm^3^ and 1.4 cm^3^. Progression to metastatic disease was observed in 2 patients (5.7%). In 8 patients (22.9%), the tumour was surgically resected, and in 8 patients (22.9%), death occurred from other causes. Two patients (5.7%) were lost to follow-up 14. We concluded that, under observation, most renal masses will grow and may require treatment. Initial tumour size cannot help to predict the natural history of renal cancer 14.

Metastasis represents the most significant risk associated with surveillance in patients with rcc because the treatment options for metastatic rcc have limited success, and thus, the disease is converted from curable to disease beyond cure.

Overall progression to metastatic disease in reported observational studies range from 0%–5.7%. In the meta-analysis by Chawla *et al*., a 1% rate of progression to metastatic disease in the renal lesions under active surveillance was noted 4. Several factors may contribute to this low rate of observed progression to metastatic disease: small cohort, relatively short duration of follow-up, and presence of benign disease in many of the observed patients.

In our recently published series (which has the longest reported follow-up to date), we also reported the highest rate of progression to metastatic disease (5.7%) 14. One patient presented with a 2.7-cm renal mass. After an initial period of observation, the patient was lost to follow-up and presented 40 months after the initial diagnosis with spinal cord compression attributable to metastatic disease. At the time of the diagnosis of metastasis, the renal mass measured 5.8 cm in the greatest dimension. Retrospectively, an annual growth rate of 0.95 cm was calculated. A second patient also presented with a 2.7-cm renal mass. An annual growth rate of 0.9 cm was observed. Given the constant size progression, the patient was offered surgical resection after 26 months of follow-up. The pathology evaluation demonstrated a conventional rcc, stage pT3aN0M0, Fuhrman grade 3. Lung metastasis and malignant pleural effusion occurred 3 months after surgery. The patient died of metastatic disease 6 months later 14. Thus, we can conclude that metastasis in srms can occur even after a long period of follow-up.

Given the risk of metastasis and the absence of curative treatment in patients with metastatic disease, we must ensure that active surveillance is offered only to appropriate patients with competing health risks. Active surveillance for srms is still lacking guidelines and cut-off values at which intervention should be considered.

A recent meta-analysis compared surveillance, nss, cryoablation, and radiofrequency ablation for the treatment of small renal lesions. A total of 99 studies were examined. The most disturbing finding was the significantly higher rates of local progression for cryoablation (rr: 7.45) and radiofrequency ablation (rr: 18.23). No difference was observed in the rates of metastatic progression between the modalities examined. The authors concluded that the current data support nss, ablation, and surveillance as viable strategies for srms based on short-term and intermediate-term oncologic outcomes. However, a significant selection bias currently exists in the clinical application of these techniques with regard to patient age and tumour size. Giving the comparable outcomes and cancer-specific survivals seen with all management modalities, the authors suggested the existence of an overtreatment bias for srms.

## 5. CONCLUSIONS

Small renal masses represent a heterogeneous disease ranging from benign to potentially highly malignant tumours. The natural history of solid renal masses is gradually being understood, and the use of surveillance as a treatment option is still being characterized.

Because no diagnostic imaging technique can predict the biologic behaviour of renal masses, the use of renal biopsy at the time of diagnosis should help in the selection of the best treatment alternative. The discovery, development, and validation of new tumour markers to help predict potential tumour growth and disease progression remain essential steps in the understanding and prediction of rcc behaviour.
